# Hemorrhagic pouchitis after bowel preparation with sodium phosphate—based enema: a case report

**DOI:** 10.1016/j.igie.2025.06.006

**Published:** 2025-07-05

**Authors:** Shanshan Wang, Huaibin M. Ko, Bo Shen

**Affiliations:** 1 The Global Integrated Center for Colorectal Surgery and IBD Interventional Endoscopy, Columbia University Irving Medical Center, New York, New York, USA; 2 Department of Pathology and Cell Biology, Columbia University Irving Medical Center, New York, New York, USA

## Abstract

Proper bowel preparation is crucial for endoscopic evaluation and intervention of the ileal pouch; yet, evidence on optimal regimens remains unclear. Although guidelines recommend the polyethylene glycol (PEG) regimens in patients with inflammatory bowel disease, sodium phosphate-based (NaP) enemas often are used for convenience. Although oral NaP has been linked to mucosal injury, similar effects from enemas have not been documented, to our knowledge. We report a case of hemorrhagic pouchitis after an NaP enema use in a patient with Crohn’s disease of the pouch who required repeated endoscopic stricturotomy. Endoscopic hemostatic agents were applied and stricture therapy was deferred to avoid further injury. No other prohemorrhagic causes besides NaP enema were identified. One month later, repeat pouchoscopy using PEG preparation showed no signs of active or recent bleeding, and endoscopic stricturotomy was successfully delivered. NaP enema should be used with caution in patients with an ileal pouch, as they can induce mucosal injury or mimic worsening pouchitis, potentially leading to misdiagnosis and inappropriate management.

## Introduction

Optimal bowel preparation is essential for ensuring diagnostic accuracy and procedural safety during endoscopy.^1^^,^^2^ The same reasoning applies to pouchoscopy, the endoscopic evaluation of ileal pouches. Various bowel preparation regimens are available, with several consensus guidelines recommending polyethylene glycol (PEG)—based preparations for patients with inflammatory bowel disease (IBD), surgically altered bowel, and those undergoing interventional lower gastrointestinal endoscopy.^2^^,^^3^ The sodium phosphate (NaP)—based enema (eg, Fleet; Prestige Consumer Healthcare Inc, Lynchburg, Va, USA), commonly used for flexible sigmoidoscopy, is also used for pouchoscopy.^3^^,^^4^ However, no data have been published regarding the optimal bowel preparation for pouchoscopy. We present a case report of hemorrhagic pouchitis resulting from NaP enema.

## Case description

**Figure 1:**
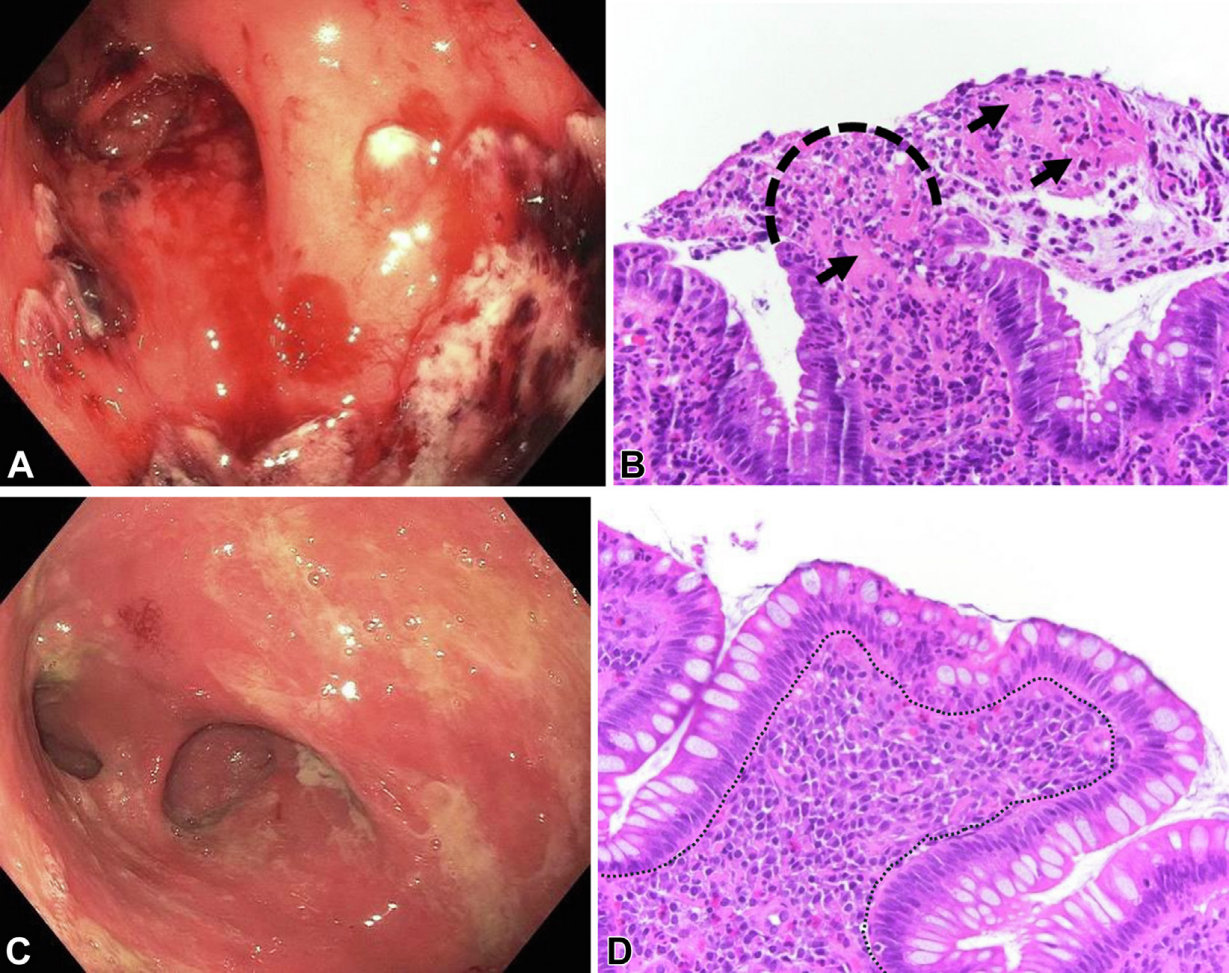
Endoscopy and histology of pouch mucosa with different bowel preparation regimens. **A,** Pouchoscopic evaluation: Edematous pouch mucosa with spontaneous bleeding from the sodium phosphate (NaP)—based enema. **B,** Histologic section of the pouch mucosa showing erosions (*black dashed line*), fibrin (*arrows*), and neutrophic exudate after NaP enema use (hematoxylin and eosin stain, ×400 magnification). **C,** Pouchoscopic evaluation: Baseline erythematous pouch mucosa with polyethylene glycol preparation. **D,** Histologic section of pouch mucosa with intact epithelium and baseline infiltration of mononuclear cells in the lamina propria (*outlined*) after polyethylene glycol preparation (hematoxylin and eosin stain, ×400 magnification).

A 64-year-old woman with Crohn’s disease of the pouch and no significant medical history presented to our Pouch Center at Columbia University Irving Medical Center for the routine endoscopic therapy of strictures. She reported no relevant family or social history. The patient had initially undergone an ileal pouch-anal anastomosis for medically refractory ulcerative colitis in 1990. J-pouch was complicated by chronic pouchitis and stricturing Crohn’s disease of the pouch, requiring treatment with long-term 6-mercaptopurine, vedolizumab, and oral vancomycin. She had multiple strictures in the anastomosis site, inlet, and prepouch ileum and had undergone serial endoscopic stricture therapy with balloon dilation and/or stricturotomy every 3 to 12 months for the last 15 years. In the most recent scheduled pouchoscopy for stricture therapy, hemorrhagic pouchitis was noted, characterized by diffuse mucosa friability and spontaneous bleeding in the pouch body (Fig. 1A), in addition to the previously mentioned strictures. Findings from the physical examination performed before the procedure were unremarkable. Histology of the pouch biopsy showed marked active inflammation, with erosions and fibrin in pouch mucosa (Fig. 1B). Bleeding was controlled with 50% dextrose (25 g/50 mL) and self-assembling peptide hydrogel spray (5 mL, PuraStat; 3-D Matrix, Newton, Mass, USA). To prevent iatrogenesis, the endoscopic stricture therapy was not delivered. We investigated the source of the bleeding. Laboratory test results revealed normal levels of platelet counts, coagulation profile, and C-reactive protein levels. The patient denied receiving pelvic radiotherapy or any antiplatelet or anticoagulant agents. It was worth noting that, in contrast to her previous PEG-based bowel preparation, she reported using 1 dose of NaP enema for the first time right before this pouchoscopy. The patient did not experience bright rectal bleeding or any other symptoms after discharge from the endoscopy unit on the same day. A conservative approach was adopted, and repeat pouchoscopy was performed 1 month later using PEG solutions for bowel preparation. Pouchoscopy revealed spontaneous improvement, with no signs of active bleeding or stigmata of recent hemorrhage, although the previously noted loss of vascularity in the pouch body persisted (Fig. 1C). The scheduled stricturomy was successfully performed without adverse events, and the patient expressed satisfaction with her management. Histologic findings were consistent with endoscopic improvement, demonstrating an intact epithelium with baseline lymphocytic infiltration of the lamina propria in pouch body (Fig. 1D).

## Discussion

We report a case of hemorrhagic pouchitis with spontaneous bleeding in a patient after bowel preparation with NaP-based enema for pouchoscopy. Although the patient had a history of chronic pouchitis and Crohn’s disease of the pouch, the spontaneous regression of pouch inflammation on both endoscopic and histologic evaluations makes progression of underlying pouch disease less likely. In addition, no prohemorrhagic or inflammatory triggers were identified.

To date, there is no solid evidence regarding optimal bowel preparations for pouchoscopy. When endoscopic interventions for the ileal pouch are planned, bowel preparation often is needed through oral regimens based on PEG, magnesium oxide, or rectal enemas based on NaP. Previous studies in patients without ileal pouch-anal anastomosis reported mucosal erosions and other inflammatory lesions associated with oral NaP-based solution at a rate of 3.3% to 24.5%,^5^, ^6^, ^7^ which could be misinterpreted as Crohn’s disease. Therefore, current guidelines favor PEG-based over NaP-based solutions for bowel preparation in patients with IBD or suspected IBD.^2^ Fleet enema, an NaP-based bowel-cleansing enema approved by the U.S. Food and Drug Administration, has established safety warnings related to kidney and heart damage if the recommended dosage (more than 1 dose in 24 hours) is exceeded.^8^ However, no bowel mucosal inflammation caused by NaP-based enema has been reported to date, to our knowledge.

Pouchoscopy is routinely performed for disease monitoring, dysplasia surveillance, and the delivery of endoscopic therapy to manage ileal pouch disorders.^3^ The feasibility of pouchoscopy is closely tied to the quality of bowel preparation. Similar to the colonic endoscopic assessment in patients with IBD, misinterpretation of the mucosal inflammation within the pouch may lead to unnecessary treatment escalation or iatrogenic adverse events during endoscopic interventions.

Endoscopic spray and rectal enema using 50% dextrose were reported to be effective in managing anastomotic bleeding, especially in circumstances where underlying tissue damage or inflammation limits the use of conventional hemostatic techniques such as endoscopic sutures or clips.^9^ In addition, self-assembling peptide hydrogel spray constitutes a promising hemostatic alternative that has been increasingly used for gastrointestinal bleeding, showing a high rate of successful hemostasis (93.1%) and a relatively low rebleeding rate (8.9%), according to a recent meta-analysis.^10^ In clinical practice, both interventions are advantageous as a result of their transparent properties, which permit direct visualization of the bleeding site during and after application. In our patient, the combined use of these 2 approaches resulted in effective hemostasis and bleeding control.

This is a case of a patient under long-term follow-up at our center, where diagnostic and therapeutic pocuhoscopy is routinely performed. The detailed understanding of her medical history and disease course prompted the clinical team to explore alternative causes of pouch bleeding beyond the common triggers of pouch inflammation: concurrent infection, fecal stasis, nonsteroidal anti-inflammatory drugs, or autoimmune disorders. However, as this represents a single case, a causal relationship between the NaP enema and hemorrhagic pouchitis can only be hypothesized, not established.

In conclusion, our case highlights the need for caution when using NaP-based enema for pouchoscopy preparation, especially in patients with suspected ongoing inflammation or scheduled therapeutic interventions.

## Data Availability Statement

The clinical data were collected from the Global Integrated Center for Colorectal Surgery and Interventional IBD and center for IBD.

## Patient Consent

The patient in this article has given written informed consent to publication of their case details.

## Disclosure

The following authors disclosed financial relationships: B. Shen: Consultant: Janssen; research/education grants: AbbVie, Takeda, and GIE Medical. All other authors disclosed no financial relationships.
